# Beyond Triple-Negative: High Prevalence of Quadruple-Negative Breast Cancer in African Americans

**DOI:** 10.3390/biomedicines12071522

**Published:** 2024-07-09

**Authors:** Oluwadamilola Oladeru, Fareed Rajack, Ashwini Esnakula, Tammey J. Naab, Yasmine Kanaan, Luisel Ricks-Santi

**Affiliations:** 1 Department of Radiation Oncology, Mayo Clinic, Jacksonville, FL 32224, USA; oladeru.oluwadamilola@mayo.edu; 2 Department of Pathology, Howard University Hospital, Washington, DC 20059, USA; rajack.fareed@gmail.com (F.R.);; 3 Department of Pathology, The Ohio State University Wexner Medical Center, Columbus, OH 43210, USA; 4 Department of Microbiology, Howard University College of Medicine, Washington, DC 20059, USA; 5 Department of Pharmacotherapy and Translational Research, University of Florida College of Pharmacy, Gainesville, FL 32610, USA

**Keywords:** quadruple-negative breast cancer, prognostic markers, triple-negative breast cancer, breast cancer, cancer disparities

## Abstract

Quadruple-negative breast cancer (QNBC) is a triple-negative breast cancer (TNBC) subtype that lacks expression of the androgen (AR) receptor. Few studies have focused on this highly aggressive breast cancer, portending worse survival rates. We aimed to determine the following: (1) QNBC’s molecular and clinical characteristics and compare them with other subtypes and (2) QNBC’s association with clinicopathological factors and prognostic markers. We performed immunohistochemical evaluations of ARs on tissue tumor microarrays from FFPE tumor blocks of invasive ductal breast carcinomas in 202 African American women. Univariate analysis was performed using the chi-square test, with survival rates calculated using Kaplan–Meier curves. Overall, 75.8% of TNBCs were AR-negative. Compared to the luminal subtypes, TNBC and QNBC tumors were likely to be a higher grade (*p* < 0.001); HER2+/AR- and QNBCs were also larger than the other subtypes (*p* < 0.001). They also expressed increasing mean levels of proteins involved in invasion, such as CD44, fascin, and vimentin, as well as decreasing the expression of proteins involved in mammary differentiation, such as GATA3 and mammaglobin. We found no association between QNBC and stage, recurrence-free survival, or overall survival rates. The high prevalence of TNBC AR-negativity in these women could explain observed worse outcomes, supporting the existence of the unique QNBC subtype.

## 1. Introduction

Breast cancer is a highly varied disease, encompassing numerous histological subtypes with distinct molecular and clinical features. Histologically, breast tumors can be described by their tissue of origin (ductal vs. lobular tissue) or by how the cells are arranged, tubule formation, nuclear grade, or mitotic count. Accurate histological characterization is crucial for treatment planning and prognosis assessment. Similarly, molecularly, breast tumors can be defined by whether or not they express estrogen receptor (ER), progesterone receptor (PR), or human epidermal growth factor receptor 2 (HER2), and among these subtypes, triple-negative breast cancer (TNBC) is defined by the absence of estrogen (ER) and progesterone (PR) receptors and human epidermal growth factor (HER2) expression. TNBC is recognized for its aggressive behavior and unfavorable prognosis and its high incidence in women of African descent [1,2]. Because ER and HER2 serve as therapeutic targets for the treatment of breast cancer, the lack thereof in TNBC remains a major challenge in the treatment of this subtype. As such, efforts have focused on the identification of novel targets that would improve breast cancer-related outcomes in patients with TNBC.

Molecular profiling has also revealed heterogeneity in TNBC, identifying potential actionable molecular targets for therapy [3]. Lehmann et al. identified at least six TNBC subtypes with specific gene expression profiles, like the basal subtype, which may have preferential responses to specific therapeutics such as cisplatin and PARP inhibitors [3,4,5,6,7]. A subtype amenable to therapeutic targeting is the Luminal Androgen Receptor (AR) subtype, also termed the apocrine subtype, which is characterized by AR signaling and androgen-dependent growth; the subtype is heavily enriched for hormonally driven pathways and is also characterized by apocrine differentiation [8,9]. While controversial, increasing evidence suggests that AR-positive TNBC may respond to therapeutic agents targeting ARs [10]. Emerging data suggest that the androgen signaling pathway plays a role in breast cancer pathogenesis and may be a valuable target using anti-androgens commonly used in the treatment of prostate cancer [11,12,13,14,15,16]. For example, Bicalutamide is an anti-androgen that blocks the effects of androgens (such as testosterone) in prostate cancer cells and helps prevent tumor growth by inhibiting androgen receptor activation. Another anti-androgen—Abiraterone Acetate—inhibits an enzyme called CYP17 that is involved in androgen synthesis, reduces androgen production, and is used in advanced prostate cancer. Therefore, understanding the clinical implications of AR expression will allow for the development of more effective and less toxic therapeutic strategies for managing patients with TNBC.

Conversely, a more recently discovered subset of TNBC, termed “quadruple-negative breast cancer” (QNBC), is characterized by the lack of androgen receptor (AR) expression [17,18,19], which is insensitive to hormonal and conventional chemotherapeutics, posing challenges for therapeutic interventions in the group [20]. This particular subtype has been reported to be disproportionately common in African American women, accounting for approximately 67% to 90% of all TNBC cases [21,22,23]. Despite its unique attributes, QNBC has received limited attention in terms of clinical and therapeutic investigations [24,25,26]. Previous studies have underscored the aggressive nature of QNBC and poorer survival outcomes compared to AR-positive breast cancer subtypes [23,25,27]. Nonetheless, our comprehension of the molecular basis of QNBC and its clinicopathological associations remains incomplete, especially in populations underrepresented in research. By examining the molecular and clinical features of QNBC in a population of African American women, a group with twice the likelihood to present with TNBC and with worse TNBC-related outcomes, this study aspires to provide valuable insights into the existing knowledge base, while stressing the necessity for additional research and tailored therapeutic approaches in this unique and understudied breast cancer subtype.

## 2. Materials and Methods

Tissue Samples. The Howard University Institutional Review Board (IRB-10-MED-24) reviewed and formally exempted this study. As previously demonstrated [28,29,30,31,32], we retrieved and coalesced a set of invasive breast ductal carcinomas (IDCs) from 202 African American women diagnosed and treated at Howard University Hospital between 2000 and 2010. Demographic and clinical information was obtained through the Howard University Cancer Center Tumor Registry and medical records. The Social Security Death Index (SSDI) was used to determine survival information for all patients. The cause of death in the SSDI is based on the National Center for Health Statistics list of 50 leading causes of death, and is converted into a three-level categorical variable of malignancy, trauma, and other.

Tissue microarrays. A series of tissue microarrays (TMAs) were constructed (Pantomics, Inc., Richmond, CA, USA) consisting of 10 × 16 arrays of 1.0 mm tissue cores from well-preserved, morphologically representative tumors in archived formalin-fixed, paraffin-embedded (FFPE) surgical blocks from 202 patients with primary IDCs. A precision tissue arrayer with two separate core needles for punching the donor and recipient blocks was used. The device also had a micrometer-precise coordinate system for tissue assembly on multi-tissue blocks. Two separate IDC tissue cores represented each surgical case in the TMA series. Each separate tissue core was assigned a unique TMA location number and was subsequently linked to an Institutional Review Board-approved database containing demographic and clinical data. Using a microtome, 5-µm sections were cut from the TMA blocks and were mounted onto Superfrost Plus microscope slides (Thermo Fisher Scientific, Waltham, MA, USA).

Immunohistochemistry. Following TMA sectioning, deparaffination was completed manually with xylene washes and serial rehydration through an alcohol–water series. Further, deparaffination, rehydration, and heat-induced antigen retrieval at pH 9.0 were performed on DAKO PT-Linker (Carpinteria, CA, USA). IHC was then completed using the DAKO Autostainer Link Chamber (Carpinteria, CA, USA) according to the manufacturer’s protocol. Antibodies utilized in this study can be found in Table 1. The binding of the primary antibody was visualized using the Avidin Biotin Complex method (ABC kit, Vector Lab). The chromogen substrate was diaminobenzidine (DAB kit, Invitrogen™ eBioscience™ DAB Advanced Chromogenic Kit, Thermo Fisher Scientific, Waltham, MA, USA). Stained slides were counterstained with hematoxylin (Invitrogen) and finally, they were treated with 70%, 95%, and absolute ethanol and xylene. Slides were cover-slipped with an automatic unit (Tissue-Tek SCA, Thermo Fisher Scientific, Waltham, MA, USA) and were examined by a pathologist under a light microscope. Nuclear AR expression was assessed using immunohistochemistry (IHC) performed on TMA sections. All sections were stained and scored by two independent observers (TN and AE) that were blinded to the clinical outcome. The sections were evaluated for the intensity of nuclear, cytoplasmic, and membranous reactivity (0–3), as well as the percentage of reactive cells (0–100%); an H-score was derived from the product of these measurements (e.g., cytoplasmic score of 2 × 30% reactive cells = H-score 60). 

Breast subtypes were defined by quantitating the nuclear immunohistochemical expression of ER (Rabbit monoclonal SP-1; 1:100), the nuclear immunohistochemical expression of PR (Mouse monoclonal PgR636; 1:500), the membranous immunohistochemical expression of HER2 (Rabbit polyclonal e-erb-2; 1:200), and the nuclear immunohistochemical expression of Ki-67 (Mouse monoclonal MIB-1; 1:100). Luminal A was characterized by a strong expression of ER or PR (H-score ≥ 200) and HER2 negativity. Luminal B was characterized by a weaker expression of ER or PR (H-score <  200) and HER2 positivity; Ki-67 > 14%; or by triple-positive expression of ER, PR, and HER2. The HER2 subtype was hormone receptor-negative with only HER2 positivity. The triple-negative subtype lacked expression of ER, PR, and HER2. Cytokeratin 5/6 immunohistochemistry staining was performed to distinguish the basal-like phenotype in the TNBCs.

Nuclear expression for AR (Mouse monoclonal Clone AR441; a:100; Agilent, Santa Clara, CA, USA) was also performed using immunohistochemistry and was considered positive if ≥10% of tumor cells showed nuclear staining. To characterize the molecular basis of QNBC, the quantitation of immunohistochemical staining for cytoplasmic cytokeratin expression (Mouse monoclonal XM26; 1:150; VWR, Radnor, PA, USA), cytoplasmic vimentin (Mouse monoclonal V9; 1:100), cytoplasmic fascin (Mouse monoclonal 55k-2; 1:500; Cell Marque Corp, Rocklin, CA, USA), nuclear GATA3 (Mouse monoclonal L50-823; 1:500; Biocare Medical, Pacheco, CA 94553, USA), and cytoplasmic mammaglobin (Mouse monoclonal 304-1A5; 1:200) was also assessed using the H-score calculation described above. These markers were chosen because of their relationship to mammary differentiation (GATA3 and mammaglobin), invasion (fascin), and epithelial-to-mesenchymal transition (Vimentin). The results were entered into a secure research database. 

Statistical Analysis. Immunohistochemical results were analyzed as continuous and categorical/bivariate variables (negative/weak and positive/moderate/strong), as described in the immunohistochemistry section. The clinicopathological variables analyzed for this study included ER status (negative or positive), PR status (negative or positive), HER2 status (negative or positive), molecular subtype (Luminal A, Luminal B, HER2+, TNBC, or QNBC), stage (I, II, III, or IV), grade (1, 2, or 3), tumor size (mm; continuous variable), overall survival rate, and recurrence-free survival rate. Univariate analysis was utilized to determine the association between TNBC, QNBC, and clinicopathological variables such as ER, PR, HER2, subtype, grade, stage, and size. As appropriate, either a chi-square test or Fisher’s exact test (if the category had a frequency of less than 5) was used to examine the association between categorical variables. In addition, ANOVA was also utilized to compare H-scores in breast tumor subtypes. Lastly, Kaplan–Meier estimates of overall survival and disease-free survival rates were plotted, and a log-rank test was performed to compare estimates among groups. All analyses were carried out using the SPSS 28 statistical program (SPSS Inc., Chicago, IL, USA). 

## 3. Results

The clinical and pathological characteristics of the study population are summarized in Table 2. Among 202 patients, 190 had data for ARs. For the 190 female patients diagnosed with invasive ductal carcinoma of the breast between 2000 and 2010, the luminal A subtype was the most frequent, constituting 43.7% of the study population. TNBC was the second most common subtype, representing 32.6% of the total number, and was purposely overrepresented to improve the study of TNBC in African American women. Notably, 75% of the TNBCs demonstrated a basal-like phenotype, which was determined using cytokeratin 5/6 immunohistochemistry. More than two-thirds of the tumors were stage I and II; however, there was a high frequency of high-grade tumors, with grade 3 tumors comprising 66.8% of the total in the study population.

**Figure 1:**
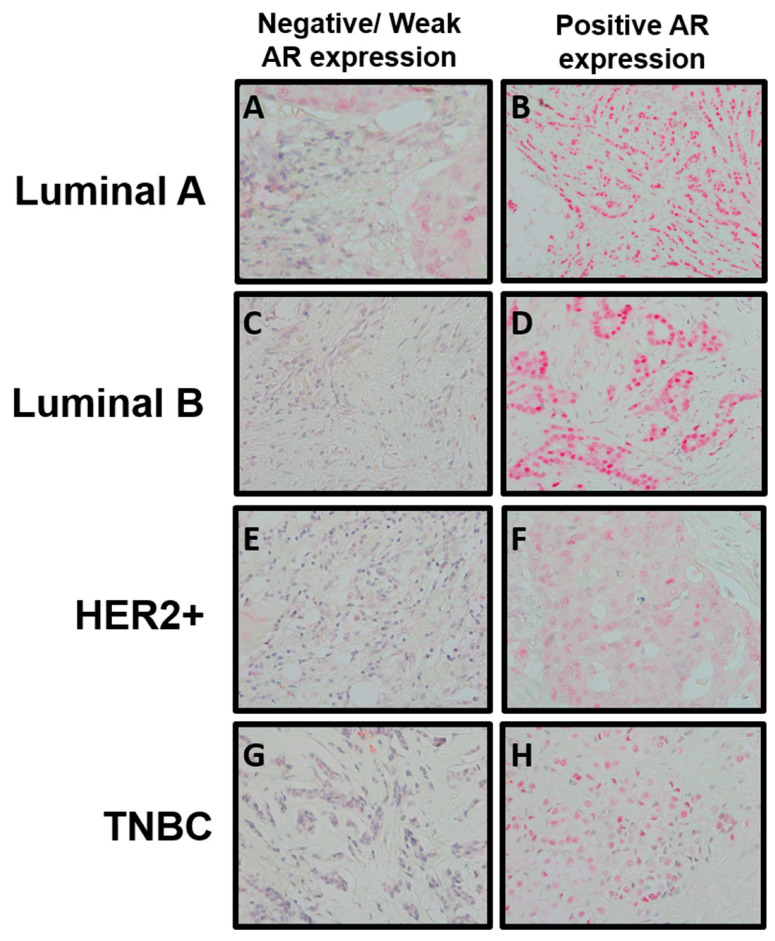
Nuclear AR expression (pink/red) in breast tumor subtypes as defined by ER, PR, and HER2 (magnification = 200×). Panels (**A**,**C**,**E**,**G**) represent negative or weak AR expression (H-score ≤ 10) in Luminal A, Luminal B, HER2+, and TNBC subtypes, respectively. In contrast, panels (**B**,**D**,**F**,**H**) represent strong nuclear expression of AR (H-score > 10) for Luminal A, Luminal B, HER2+ and TNBC subtypes, respectively.

**Figure 2:**
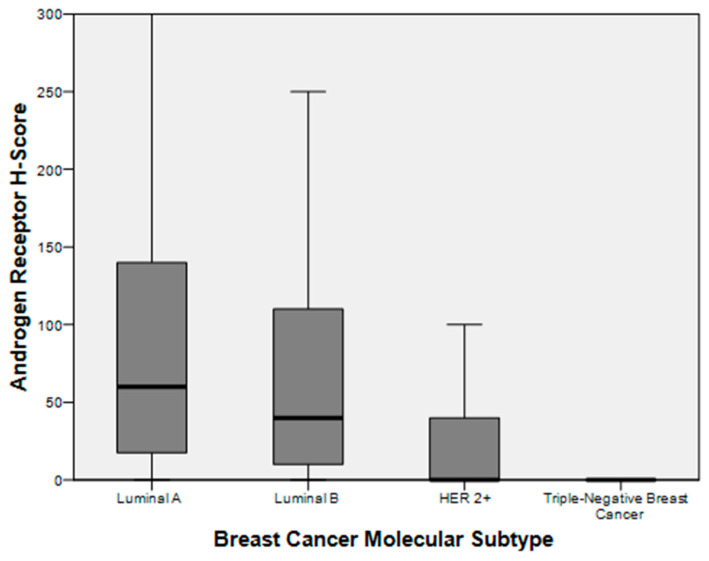
H-scores for AR IHC by molecular subtype. Luminal A tumors have the highest median AR expression, followed by luminal B, HER2+ tumors, and TNBC. TNBCs demonstrate heterogeneity with outlying tumors strongly expressing AR.

Of the luminal A, luminal B, HER2-overexpressing, and TNBC subtypes, 20.2%, 22.2%, 55.6%, and 75.8% were AR-negative (*p* < 0.001). Images of the AR expression in each breast cancer subtype are depicted in Figure 1. AR expression and H-scores were also plotted and were found to be the highest for luminal A tumors (mean = 92.23 ± 87.11), followed by luminal B tumors (mean = 69.15 ± 79.24) and HER2-overexpressing tumors (mean = 32.06 ± 61.10). TNBC had the lowest AR H-score with a mean of 21.89 (±55.45) (ANOVA *p*  <  0.001) (Figure 2).

Compared to the luminal subtypes, TNBC and QNBC had a higher frequency of grade 3 tumors (*p* < 0.001) (Table 3). When subtypes were stratified by AR status, the HER2-overexpressing tumors that were AR-negative were the largest (mean = 64.30 mm ± 44.91 SD), followed by QNBCs (mean = 37.31 mm ± 29.36 SD) and luminal A/AR-negative tumors (mean = 32.87 mm ± 24.40 SD) (Table 4). There were no associations with stage, metastases, lymph node positivity, or recurrence (Table 3). Increasing age was also associated with AR positivity (Pearson’s correlation = 0.15, *p* =  0.037).

**Figure 3:**
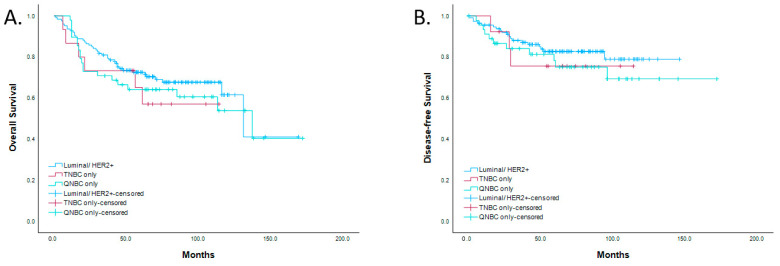
Survival analysis of luminal tumors, TNBC, and QNBC. No differences in (**A**) verall survival and (**B**) disease-free survival were noted between luminal tumors, TNBC, and QNBC.

QNBC or TNBC status was not associated with overall or disease-free survival rates (Figure 3). Compared to luminal tumors and TNBCs, QNBCs had a higher mean expression of markers associated with invasion (Fascin, *p* <  0.001) and reduced survival markers (vimentin, *p*  <  0.001) (Table 5). QNBCs also had a lower mean expression of (1) GATA3 (*p* <  0.001), a regulator of mammary luminal cell differentiation and an ER-associated marker in breast cancer, and (2) mammaglobin (*p*  =  0.008), another protein involved in mammary cell differentiation (Table 5).

## 4. Discussion

TNBCs, accounting for 15–20% of all breast carcinomas, lack ER, PR, and HER2 expression and are frequently high-grade, large at diagnosis, and have high recurrence rates. Still, TNBC is a complex disease, and ongoing research aims to improve therapeutic options and patient outcomes. TNBC has a worse prognosis compared to hormone receptor (HR)-positive breast cancers due to its aggressive nature and the lack of therapeutic options, with standard treatment options being limited to surgery, adjuvant chemotherapy, and radiotherapy. For example, the basal-like 1 (BL1) subtype is characterized by high proliferation rates and DNA repair deficiency. Basal-like 2 (BL2) tumors exhibit immune activation and are associated with a better prognosis compared to other basal-like subtypes. Basal-like tumors respond well to chemotherapy but have a higher risk of recurrence. PARP inhibitors, which target DNA repair deficiency have shown promise in clinical trials for metastatic TNBC and other types of tumors [33,34]. Like BL2s, immunomodulatory (IM) tumors exhibit immune activation, are associated with a better prognosis, and respond well to immunotherapies. Checkpoint inhibitors (e.g., PD-1/PD-L1 inhibitors) have improved overall survival and response in these types of TNBCs [35]. Conversely, basal-like immune-suppressed tumors have a low immune activity and are less responsive to immunotherapy. The Mesenchymal (M) and Mesenchymal-like (MSL) tumors are highly aggressive and are associated with poor outcomes. Finally, luminal AR (LAR) tumors express AR and have a luminal-like gene expression profile. They are less responsive to chemotherapy but may benefit from anti-androgen therapies.

Like the subtypes listed above, QNBC represents a subtype of breast cancer characterized by the lack of expression of ERs, PRs, HER2, and ARs. This subtype has emerged as a distinct entity within the broader category of TNBC. However, few studies on QNBC published thus far have investigated this subtype’s clinical significance, prognostic factors, molecular features, and treatment response. The studies highlight the association of QNBC with high genomic instability, copy number alterations, and miRNA deregulation [36]. They also report the expression of ARs in a subset of TNBCs and suggest that AR status may have a role in predicting prognosis and therapeutic response [27,37,38,39,40,41,42,43,44,45,46,47,48]. A few reviews discuss the molecular subtypes of breast cancer, including QNBC, and their clinical implications [18,19,20]. Some studies found that AR-positive TNBCs have a better prognosis and survival rate compared to QNBCs [23,24,49,50,51,52,53]. Additionally, a few studies have explored the potential role of AR expression in predicting the response to neoadjuvant chemotherapy in TNBC [25,47]. 

In our study, we aimed to investigate the molecular and clinical characteristics of QNBC and their associations with clinicopathological factors and prognostic markers in a cohort of 190 African American women diagnosed with invasive ductal carcinoma. Our results provide valuable insights into the distinct features of QNBC and emphasize the need for further research and the development of targeted therapeutic strategies for this unique breast cancer subtype. We found that 75.8% of TNBC cases were AR-negative and reflected a high prevalence of QNBC in African American women [22]. The tumors in our cohort were predominantly high-grade, which is consistent with previous reports of a higher frequency of aggressive breast cancer subtypes in African American women. HER2+/AR− and QNBCs were larger in size, with QNBC having the highest grade tumors compared to other subtypes.

However, our analysis did not reveal any significant correlations between QNBC and variables such as stage, metastasis, lymph node positivity, or recurrence, which may be attributable to the limited sample size, potentially constraining our capacity to identify differences. However, QNBCs demonstrated a higher expression of markers associated with invasion (fascin) and reduced survival rates (vimentin) compared to luminal tumors and TNBCs. These results suggest that QNBC may have a more aggressive biology, which could contribute to the worse clinical outcomes observed in this subtype. Additionally, QNBCs displayed lower levels of GATA3 and mammaglobin—both of which play significant roles in cell differentiation, proliferation, and mammary cell differentiation. The downregulation of these genes could further explain the aggressive nature of QNBC and its resistance to conventional therapies.

Despite the associations between QNBC and aggressive clinicopathological features, our cohort study did not find a significant association between QNBC status and overall survival or disease-free survival rates. This could be attributed to several factors, including the relatively small sample size, the retrospective nature of the study, or other confounding factors not accounted for in our analysis. Future prospective studies with larger cohorts, ancestry data, and long-term follow-up data are needed to validate and expand upon these findings.

## 5. Conclusions

In summary, our comprehensive investigation into quadruple-negative breast cancer (QNBC) among African American women has illuminated critical insights into this aggressive and understudied subtype. The distinct molecular and clinical features of QNBC underscore the urgency of further research to unravel the mechanisms driving its behavior. By deepening our understanding of QNBC, we pave the way for novel therapeutic strategies that can specifically target this challenging cancer variant. Moreover, the disproportionate prevalence of QNBC in African American women demands a concerted effort to address disparities in breast cancer research and treatment. Bridging these gaps is essential to improving outcomes and ensuring equitable access to effective therapies for this vulnerable population. As we continue our scientific journey, collaboration, advocacy, and targeted interventions will be pivotal in transforming the landscape of QNBC management and ultimately saving lives.

## Figures and Tables

**Table 1:** List of the antibodies utilized in the study and characteristics of the corresponding dilution of the specific anti-human antibody used in the immunohistochemistry study. Staining characteristic: expression location in the cell (nuclear, membranous, cytoplasmic).

Markers	Antibodies	Clone	Dilution	Source	Expression
Estrogen Receptor	Rabbit monoclonal	SP-1	1:100	Dako	Nuclear
Progesterone Receptor	Mouse monoclonal	PgR636	1:500	Dako	Nuclear
Human Epidermal Growth Factor Receptor 2	Rabbit polyclonal	e-erb-2	1:200	Dako	Membranous
Cytokeratin 5	Mouse monoclonal	XM26	1:150	VWR	Cytoplasmic
Ki-67	Mouse monoclonal	MIB-1	1:100	Dako	Nuclear
Vimentin	Mouse monoclonal	V9	1:100	Dako	Cytoplasmic
Fascin	Mouse monoclonal	55k-2	1:500	Cell Marque Corp	Cytoplasmic
GATA3	Mouse monoclonal	L50–823	1:500	Biocare Medical	Nuclear
Mammaglobin	Mouse monoclonal	304–1A5	1:200	Dako	Cytoplasmic
Androgen Receptor	Mouse monoclonal	Clone AR441	1:100	Agilent	Nuclear

**Table 2:** Clinical and pathological characteristics of study population.

Characteristic	Frequency (%) *n* = 190
Age, years	
<50	54 (28.4)
≥50	136 (71.6)
ER status	
Positive	109 (57.4)
Negative	81 42.6)
PR status	
Positive	91 (47.9)
Negative	99 (52.1)
Subtype ^a^	
Luminal A	83 (43.7)
Luminal B	27 (14.2)
HER2+	18 (9.5)
Triple-negative	62 (32.6)
Pathologic stage	
Stage I	59 (31.1)
Stage II	79 (41.6)
Stage III	40 (21.1)
Stage IV	12 (6.3)
Grade	
Grade 1	9 (4.7)
Grade 2	54 (28.4)
Grade 3	127 (66.8)
Recurrence	
None	128 (67.4)
Loco-regional	10 (5.3)
Distant	20 (10.5)
Never disease-free	17 (8.9)
Unknown	15 (7.9)

^a^ Luminal A: ER+ or PR+, HER2+; luminal B: ER+ or PR+, HER2+; HER2: ER−, PR−, HER2+; triple-negative: ER−, PR−, HER2+; Abbreviations: ER, estrogen receptor; PR, progesterone receptor; HER2+, human epidermal growth factor receptor 2.

**Table 3:** Summary of associations with clinicopathological features.

		Luminal A (%)		Luminal B (%)		HER2+ (%)		TNBC (%)		QNBC (%)		*p*-Value
		*n* = 83		*n* = 14		*n* = 18		*n* = 43		*n* = 14		
Stage												0.378
	Stage I	27	32.5%	11	40.7%	3	16.7%	15	31.9%	3	20.0%	
	Stage II	37	44.6%	7	25.9%	6	33.3%	19	40.4%	10	66.7%	
	Stage III	15	18.1%	7	25.9%	7	38.9%	10	21.3%	1	6.7%	
	Stage IV	4	4.8%	2	7.4%	2	11.1%	3	6.4%	1	6.7%	
Grade												<0.001
	Grade 1	5	6.0%	3	11.1%	0	0.0%	0	0.0%	0	0.0%	
	Grade 2	39	47.0%	10	37.0%	0	0.0%	4	8.5%	1	6.7%	
	Grade 3	39	47.0%	14	51.9%	18	100.0%	43	91.5%	14	93.3%	
Distant Metastases												0.242
	No	75	90.4%	20	74.1%	14	77.8%	38	80.9%	13	86.7%	
	Yes	8	9.6%	7	25.9%	4	22.2%	9	19.1%	2	13.3%	
Lymph Nodes												0.759
	No	43	59.7%	13	52.0%	7	43.8%	24	60.0%	8	61.5%	
	Yes	29	40.3%	12	48.0%	9	56.3%	16	40.0%	5	38.5%	
	Missing	11		2		2		7		2		
Recurrence												0.425
	None	65	87.8%	20	74.1%	13	81.3%	36	76.6%	10	76.9%	
	Yes	9	12.2%	7	25.9%	3	18.8%	11	23.4%	3	23.1%	
	Missing	9				2		0		2		

Abbreviations: TNBC, triple-negative breast cancer; QNBC, quadruple-negative breast cancer.

**Table 4:** Mean size and standard deviation for subtypes with AR status.

Subtype/AR Status	Mean (mm)	N	SD	*p*-Value
Luminal A/AR negative	32.87	15	24.40	<0.001
Luminal A/AR positive	24.47	60	15.65	
Luminal B/AR negative	22.00	6	20.00	
Luminal B/AR positive	31.37	19	21.50	
HER2+/AR negative	64.30	10	44.91	
HER2+/AR positive	27.88	8	14.00	
Quadruple negative BC	37.31	42	29.36	
Triplenegative BC	25.36	14	13.09	

**Table 5:** Markers differentially expressed in TNBC and QNBC tumors.

		Luminal Subtype		TNBC		QNBC			
		Mean	Std Dev	Mean	Std Dev	Mean	Std Dev	F Statistic	ANOVA *p*-Value
Mammary differentiation/tumor suppressor	GATA3	216.82	76.84	81.15	87.99	15.74	48.05	138.71	<0.001
Mammary differentiation/tumor suppressor	Mammaglobin	98.96	110.76	58.46	101.87	46.21	63.86	5.00	0.008
Invasiveness/oncogene	Fascin	11.34	39.06	97.67	113.21	178.80	101.94	99.89	<0.001
EMT marker/oncogene	Vimentin	12.84	57.29	64.00	105.83	117.66	104.49	30.69	<0.001

Abbreviations: TNBC, triple-negative breast cancer; QNBC, quadruple-negative breast cancer; Std Dev, standard deviation; F, frequency.

## Data Availability

The authors agree to share anonymized data upon reasonable request by researchers.

## References

[1] DeSantis C.E., Ma J., Goding Sauer A., Newman L.A., Jemal A. (2017). Breast cancer statistics, 2017, racial disparity in mortality by state. CA Cancer J. Clin..

[2] Lund M.J., Trivers K.F., Porter P.L., Coates R.J., Leyland-Jones B., Brawley O.W., Flagg E.W., O’Regan R.M., Gabram S.G., Eley J.W. (2009). Race and triple negative threats to breast cancer survival: A population-based study in Atlanta, GA. Breast Cancer Res. Treat..

[3] Lehmann B.D., Bauer J.A., Chen X., Sanders M.E., Chakravarthy A.B., Shyr Y., Pietenpol J.A. (2011). Identification of human triple-negative breast cancer subtypes and preclinical models for selection of targeted therapies. J. Clin. Investig..

[4] Isakoff S.J., Mayer E.L., He L., Traina T.A., Carey L.A., Krag K.J., Rugo H.S., Liu M.C., Stearns V., Come S.E. (2015). TBCRC009: A Multicenter Phase II Clinical Trial of Platinum Monotherapy with Biomarker Assessment in Metastatic Triple-Negative Breast Cancer. J. Clin. Oncol..

[5] Li Q., Li Q., Zhang P., Yuan P., Wang J., Ma F., Luo Y., Fan Y., Cai R., Xu B. (2015). A phase II study of capecitabine plus cisplatin in metastatic triple-negative breast cancer patients pretreated with anthracyclines and taxanes. Cancer Biol. Ther..

[6] Min A., Im S.A., Kim D.K., Song S.H., Kim H.J., Lee K.H., Kim T.Y., Han S.W., Oh D.Y., Kim T.Y. (2015). Histone deacetylase inhibitor, suberoylanilide hydroxamic acid (SAHA), enhances anti-tumor effects of the poly (ADP-ribose) polymerase (PARP) inhibitor olaparib in triple-negative breast cancer cells. Breast Cancer Res..

[7] Gross E., van Tinteren H., Li Z., Raab S., Meul C., Avril S., Laddach N., Aubele M., Propping C., Gkazepis A. (2016). Identification of BRCA1-like triple-negative breast cancers by quantitative multiplex-ligation-dependent probe amplification (MLPA) analysis of BRCA1-associated chromosomal regions: A validation study. BMC Cancer.

[8] Gucalp A., Traina T.A. (2017). Androgen receptor-positive, triple-negative breast cancer. Cancer.

[9] Meligy B.M., Tawfik E.A., El Khouly E.A., Alagizy H., Shehata M.A., Elkady N.M. (2003). Androgen receptor expression in estrogen receptor-negative breast cancer. Appl. Immunohistochem. Mol. Morphol..

[10] Ferrari P., Scatena C., Ghilli M., Bargagna I., Lorenzini G., Nicolini A. (2022). Molecular Mechanisms, Biomarkers and Emerging Therapies for Chemotherapy Resistant TNBC. Int. J. Mol. Sci..

[11] Astvatsaturyan K., Yue Y., Walts A.E., Bose S. (2018). Androgen receptor positive triple negative breast cancer: Clinicopathologic, prognostic, and predictive features. PLoS ONE.

[12] Rampurwala M., Wisinski K.B., O’Regan R. (2016). Role of the androgen receptor in triple-negative breast cancer. Clin. Adv. Hematol. Oncol..

[13] Bianchini G., Balko J.M., Mayer I.A., Sanders M.E., Gianni L. (2016). Triple-negative breast cancer: Challenges and opportunities of a heterogeneous disease. Nat. Rev. Clin. Oncol..

[14] Narayanan R., Dalton J.T. (2016). Androgen Receptor: A Complex Therapeutic Target for Breast Cancer. Cancers.

[15] Gerratana L., Basile D., Buono G., De Placido S., Giuliano M., Minichillo S., Coinu A., Martorana F., De Santo I., Del Mastro L. (2018). Androgen receptor in triple negative breast cancer: A potential target for the targetless subtype. Cancer Treat. Rev..

[16] You C.P., Leung M.H., Tsang W.C., Khoo U.S., Tsoi H. (2022). Androgen Receptor as an Emerging Feasible Biomarker for Breast Cancer. Biomolecules.

[17] Tsutsumi Y. (2012). Apocrine carcinoma as triple-negative breast cancer: Novel definition of apocrine-type carcinoma as estrogen/progesterone receptor-negative and androgen receptor-positive invasive ductal carcinoma. Jpn. J. Clin. Oncol..

[18] Barton V.N., D’Amato N.C., Gordon M.A., Christenson J.L., Elias A., Richer J.K. (2015). Androgen Receptor Biology in Triple Negative Breast Cancer: A Case for Classification as AR+ or Quadruple Negative Disease. Horm. Cancer.

[19] Hon J.D., Singh B., Sahin A., Du G., Wang J., Wang V.Y., Deng F.M., Zhang D.Y., Monaco M.E., Lee P. (2016). Breast cancer molecular subtypes: From TNBC to QNBC. Am. J. Cancer Res..

[20] Huang M., Wu J., Ling R., Li N. (2020). Quadruple negative breast cancer. Breast Cancer.

[21] Haruna M., Daramola A.O., Awolola N.A., Badr N.M., Banjo A.A.F., Shaaban A. (2022). Clinicopathological features and androgen receptor expression in triple negative breast cancer at Lagos, Nigeria. Ecancermedicalscience.

[22] Jinna N., Jovanovic-Talisman T., LaBarge M., Natarajan R., Kittles R., Sistrunk C., Rida P., Seewaldt V.L. (2022). Racial Disparity in Quadruple Negative Breast Cancer: Aggressive Biology and Potential Therapeutic Targeting and Prevention. Cancers.

[23] Davis M., Tripathi S., Hughley R., He Q., Bae S., Karanam B., Martini R., Newman L., Colomb W., Grizzle W. (2018). AR negative triple negative or “quadruple negative” breast cancers in African American women have an enriched basal and immune signature. PLoS ONE.

[24] Christenson J.L., Trepel J.B., Ali H.Y., Lee S., Eisner J.R., Baskin-Bey E.S., Elias A.D., Richer J.K. (2018). Harnessing a Different Dependency: How to Identify and Target Androgen Receptor-Positive versus Quadruple-Negative Breast Cancer. Horm. Cancer.

[25] Mohammed A.A., Elsayed F.M., Algazar M., Rashed H.E., Anter A.H. (2020). Neoadjuvant Chemotherapy in Triple Negative Breast Cancer: Correlation between Androgen Receptor Expression and Pathological Response. Asian Pac. J. Cancer Prev..

[26] Azim H.A., Shohdy K.S., Elghazawy H., Salib M.M., Almeldin D., Kassem L. (2022). Programmed death-ligand 1 (PD-L1) expression predicts response to neoadjuvant chemotherapy in triple-negative breast cancer: A systematic review and meta-analysis. Biomarkers.

[27] Riaz N., Idress R., Habib S., Lalani E.N. (2020). Lack of Androgen Receptor Expression Selects for Basal-like Phenotype and Is a Predictor of Poor Clinical Outcome in Non-Metastatic Triple Negative Breast Cancer. Front. Oncol..

[28] Ricks-Santi L.J., Fredenburg K., Rajaei M., Esnakula A., Naab T., McDonald J.T., Kanaan Y. (2023). Characterization of GATA3 and Mammaglobin in breast tumors from African American Women. Arch. Microbiol. Immunol..

[29] Beyene D., Naab T., Apprey V., Ricks-Santi L., Esnakula A., Qasim M., George M., Minoza K.G., Copeland R.L., Broome C. (2023). Cyclin A2 and Ki-67 proliferation markers could be used to identify tumors with poor prognosis in African American women with breast cancer. J. Cancer Biol..

[30] Khan F., Ricks-Santi L.J., Zafar R., Kanaan Y., Naab T. (2018). Expression of p27 and c-Myc by immunohistochemistry in breast ductal cancers in African American women. Ann. Diagn. Pathol..

[31] Khan F., Esnakula A., Ricks-Santi L.J., Zafar R., Kanaan Y., Naab T. (2018). Loss of PTEN in high grade advanced stage triple negative breast ductal cancers in African American women. Pathol. Res. Pract..

[32] Esnakula A.K., Ricks-Santi L., Kwagyan J., Kanaan Y.M., DeWitty R.L., Wilson L.L., Gold B., Frederick W.A., Naab T.J. (2014). Strong association of fascin expression with triple negative breast cancer and basal-like phenotype in African-American women. J. Clin. Pathol..

[33] Maqbool M., Bekele F., Fekadu G. (2022). Treatment Strategies Against Triple-Negative Breast Cancer: An Updated Review. Breast Cancer Targets Ther..

[34] Mandapati A., Lukong K.E. (2023). Triple negative breast cancer: Approved treatment options and their mechanisms of action. J. Cancer Res. Clin. Oncol..

[35] Bhattarai S., Sugita B.M., Bortoletto S.M., Fonseca A.S., Cavalli L.R., Aneja R. (2021). QNBC Is Associated with High Genomic Instability Characterized by Copy Number Alterations and miRNA Deregulation. Int. J. Mol. Sci..

[36] Di Leone A., Fragomeni S.M., Scardina L., Ionta L., Mule A., Magno S., Terribile D., Masetti R., Franceschini G. (2021). Androgen receptor expression and outcome of neoadjuvant chemotherapy in triple-negative breast cancer. Eur. Rev. Med. Pharmacol. Sci..

[37] Xu M., Yuan Y., Yan P., Jiang J., Ma P., Niu X., Ma S., Cai H., Yang K. (2020). Prognostic Significance of Androgen Receptor Expression in Triple Negative Breast Cancer: A Systematic Review and Meta-Analysis. Clin. Breast Cancer.

[38] Naimi A., Soltan M., Amjadi E., Goli P., Kefayat A. (2020). Androgen Receptor Expression and Its Correlation with Clinicopathological Parameters in Iranian Patients with Triple Negative Breast Cancer. Iran. J. Pathol..

[39] Zuo T., Wilson P., Cicek A.F., Harigopal M. (2018). Androgen receptor expression is a favorable prognostic factor in triple-negative breast cancers. Hum. Pathol..

[40] Sunar V., Dogan H.T., Sarici F., Ates O., Akin S., Baspinar B., Aksoy S., Altundag K. (2018). Association between androgen receptor status and prognosis in triple negative breast cancer. J. BUON.

[41] Asano Y., Kashiwagi S., Goto W., Tanaka S., Morisaki T., Takashima T., Noda S., Onoda N., Ohsawa M., Hirakawa K. (2017). Expression and Clinical Significance of Androgen Receptor in Triple-Negative Breast Cancer. Cancers.

[42] Maeda T., Nakanishi Y., Hirotani Y., Fuchinoue F., Enomoto K., Sakurai K., Amano S., Nemoto N. (2016). Immunohistochemical co-expression status of cytokeratin 5/6, androgen receptor, and p53 as prognostic factors of adjuvant chemotherapy for triple negative breast cancer. Med. Mol. Morphol..

[43] Choi J.E., Kang S.H., Lee S.J., Bae Y.K. (2015). Androgen receptor expression predicts decreased survival in early stage triple-negative breast cancer. Ann. Surg. Oncol..

[44] Pistelli M., Caramanti M., Biscotti T., Santinelli A., Pagliacci A., De Lisa M., Ballatore Z., Ridolfi F., Maccaroni E., Bracci R. (2014). Androgen receptor expression in early triple-negative breast cancer: Clinical significance and prognostic associations. Cancers.

[45] Tang D., Xu S., Zhang Q., Zhao W. (2012). The expression and clinical significance of the androgen receptor and E-cadherin in triple-negative breast cancer. Med. Oncol..

[46] He J., Peng R., Yuan Z., Wang S., Peng J., Lin G., Jiang X., Qin T. (2012). Prognostic value of androgen receptor expression in operable triple-negative breast cancer: A retrospective analysis based on a tissue microarray. Med. Oncol..

[47] Loibl S., Muller B.M., von Minckwitz G., Schwabe M., Roller M., Darb-Esfahani S., Ataseven B., du Bois A., Fissler-Eckhoff A., Gerber B. (2011). Androgen receptor expression in primary breast cancer and its predictive and prognostic value in patients treated with neoadjuvant chemotherapy. Breast Cancer Res. Treat..

[48] Bhattarai S., Saini G., Gogineni K., Aneja R. (2020). Quadruple-negative breast cancer: Novel implications for a new disease. Breast Cancer Res..

[49] Angajala A., Mothershed E., Davis M.B., Tripathi S., He Q., Bedi D., Dean-Colomb W., Yates C. (2019). Quadruple Negative Breast Cancers (QNBC) Demonstrate Subtype Consistency among Primary and Recurrent or Metastatic Breast Cancer. Transl. Oncol..

[50] Safarpour D., Pakneshan S., Tavassoli F.A. (2014). Androgen receptor (AR) expression in 400 breast carcinomas: Is routine AR assessment justified?. Am. J. Cancer Res..

[51] McGhan L.J., McCullough A.E., Protheroe C.A., Dueck A.C., Lee J.J., Nunez-Nateras R., Castle E.P., Gray R.J., Wasif N., Goetz M.P. (2014). Androgen receptor-positive triple negative breast cancer: A unique breast cancer subtype. Ann. Surg. Oncol..

[52] Mrklic I., Pogorelic Z., Capkun V., Tomic S. (2013). Expression of androgen receptors in triple negative breast carcinomas. Acta Histochem..

[53] Choi Y.L., Oh E., Park S., Kim Y., Park Y.H., Song K., Cho E.Y., Hong Y.C., Choi J.S., Lee J.E. (2010). Triple-negative, basal-like, and quintuple-negative breast cancers: Better prediction model for survival. BMC Cancer.

